# Chinese cabbage orphan gene *BR3* confers bolting resistance to *Arabidopsis* through the gibberellin pathway

**DOI:** 10.3389/fpls.2024.1518962

**Published:** 2025-01-20

**Authors:** Yuting Zhang, Mingliang Jiang, Shurui Sun, Zongxiang Zhan, Xiaonan Li, Zhongyun Piao

**Affiliations:** ^1^ Molecular Biology of Vegetable Laboratory, College of Horticulture, Shenyang Agricultural University, Shenyang, China; ^2^ School of Agriculture, Jilin Agricultural Science and Technology University, Jilin, China

**Keywords:** Chinese cabbage, orphan gene, *BR3*, bolting resistance, *Arabidopsis*, GA pathway

## Abstract

Premature bolting reduces the yield and quality of Chinese cabbage, making bolting resistance gene identification crucial for breeding superior and stable varieties. In this study, we identified an orphan gene *BOLTING RESISTANCE 3* (*BR3*) that positively regulates bolting resistance in *Arabidopsis thaliana*. The expression of *BR3* was developmentally regulated and occurred during the seedling and flowering stages. The BR3 protein was localized to both the plasma membrane and nucleus. *Arabidopsis BR3* overexpressing (*BR3*OE) plants exhibited delayed bolting and flowering times, an increased number of rosette leaves, reduced plant height, and fewer siliques under long-day (LD) conditions. Key flowering genes were significantly downregulated in *BR3*OE plants. *BR3*OE plants similarly exhibited delayed bolting and flowering times, and an increased number of rosette leaves under short-day (SD) conditions. *BR3*OE plants showed no significant phenotypic differences after vernalization treatment. *BR3*OE and WT plants exhibited early flowering after GA_3_ treatment, and bolting and flowering time remained delayed in *BR3*OE plants compared with WT plants. Key *DELLA* genes *BrRGA1* and *BrRGL3* exhibited a co-expression pattern consistent with *BR3* gene in Chinese cabbage, which suggested that *BrRGA1* and *BrRGL3* genes may directly or indirectly regulated by *BR3* gene. *BR3* gene increased bolting resistance perhaps by upregulating the expression of *DELLA* genes in the GA pathway. This study provides new theoretical insights for addressing premature bolting in Chinese cabbage and offers novel approaches for breeding bolting-resistant varieties.

## 1 Introduction

Orphan genes (*OGs*) are widely present in every species and have no significant sequence similarity to known genes (Li and Wurtele, 2014; Rdelsperger et al., 2019; Jiang et al., 2022). Numerous plant genomes have been rapidly decoded with the sequencing technology advancements, which provided a solid foundation for identifying *OGs*. A number of *OGs* have been identified in diverse species. For instance, there are 1324 *OGs* in the genome of *Arabidopsis* and 529 in the genome of *B. rapa* (Lin et al., 2010; Jiang et al., 2018). These genes lack recognizable functional domains, motifs, or structures, posing significant challenges for functional characterization of *OGs*. However, previous studies have shown that *OGs* play crucial roles in biotic and abiotic stress responses (Luhua et al., 2008; Jiang et al., 2018; Qi et al., 2018; Li et al., 2019; Jiang et al., 2020a; Wang et al., 2021; Tanvir et al., 2022), metabolism regulation (Li and Wurtele, 2014; Li et al., 2015; Jones et al., 2016; Jiang et al., 2020b; Wang et al., 2024), and species-specific trait formation (Hanada et al., 2008; Cui et al., 2015; Ni et al., 2017; O’Conner and Li, 2020; Jiang et al., 2023; Zu et al., 2024). The functions of *OGs* in plant growth and development recently garnered increasing attention. The interaction of *Arabidopsis* ICE1 (INDUCER OF CBF EXPRESSION 1) and IDD14 (INDETERMINATE DOMAIN 14) activates the transcription of *OGs* to regulate lipid metabolism in pollen, thus promoting pollen development and viability (Luo et al., 2024). Additionally, a novel *OG*, *Bolting Resistance 1* (*BR1*), has been identified as a bolting resistance regulator in *B. rapa*, specifically delay flowering through vernalization and photoperiod pathways (Jiang et al., 2023). *OG Bolting Resistance 2* (*BR2*) that regulates bolting resistance through the vernalization pathway, and its *Arabidopsis* overexpression upregulated flowering repressor *FLC* and downregulated key floral integrators (Zu et al., 2024). These findings highlight the vital roles of Chinese cabbage *OGs* in bolting resistance, although the exact mechanisms remain unclear.

Flowering time is a crucial agronomic trait of plant growth and development that is influenced by external environmental signals (e.g., photoperiod, temperature, and vernalization) and internal factors (e.g., autonomous pathways, age, and GA) (Pieper et al., 2020). Hormones, particularly GAs, are involved in cell division, elongation, and the transition from seed germination to flowering (Macmillan and Takahashi, 1968; Teotia and Tang, 2015). GAs, a class of diterpenoid plant hormones, promote flowering upon appropriate exogenous application (Hedden, 2020; Zhang et al., 2020). Defects in GA biosynthesis and signaling pathways often lead to aberrant flowering phenotypes, such as in GA-deficient mutant *ga1-3*, which does not flower under SD conditions (Wilson et al., 1992). Conversely, *SPINDLY* (*SPY*) is a negative regulator of the GA signaling pathway, and the enhancement of GA signaling in *spy* mutants leads to early flowering in *Arabidopsis* (Silverstone et al., 2006). As central GA signaling components, DELLA proteins inhibit flowering by interacting with the BRM-NF-YC functional module (Zhang et al., 2023). DELLA proteins delay flowering by repressing the expression of flowering-promoting factors, such as *SOC1* and *LFY*. When GA signaling is enhanced, DELLA proteins are degraded, thereby relieving the repression of these genes (Achard and Genschik, 2008). Transcription factor *WRKY75* regulates the GA signaling pathway by interacting with DELLA proteins, thus influencing flowering time and the photoperiod response in *A. thaliana* (Zhang et al., 2017). Recent research has shown that several regulatory factors influence GA signaling through distinct mechanisms, including *C-TERMINAL DOMAIN PHOSPHATASE-LIKE3* (*CPL3*), *Basic helix-loop-helix 4* (*MdbHLH4*), *D2-Hydroxyglutarate Dehydroase* (*GhD2HGDH*), and *KNOTTED-like homeobox 15* (*MdKNOX15*). Although the role of GA signaling in flowering time regulation has been widely studied, its precise molecular mechanisms remain to be elucidated.

In this study, a novel *B. rapa OG BR3* was identified. The expression patterns and subcellular localization of *BR3* were determined. Flowering time and other related traits of *A. thaliana BR3*OE plants were analyzed under LD, SD, vernalization, and GA_3_ treatments. Additionally, the expression patterns of key flowering-related genes were determined using qRT-PCR analysis. This study evaluated the specific pathway through which *BR3* regulates flowering, providing new insights into the function of *OGs* and offering a novel approach for breeding bolting-resistant Chinese cabbage varieties.

## 2 Materials and methods

### 2.1 Plant materials and cultivation

The plant materials used in this study were Chinese cabbage inbred line ‘GT-24’, wild-type *A. thaliana* (WT), T_3_ generation of *BR3*-overexpressing *Arabidopsis* plants (‘*BR3*OE’), and cultivated *Nicotiana benthamiana*. The cultivation methods followed those described in a previous study (Jiang et al., 2023).

### 2.2 *BR3* sequence analysis, vector construction, and plant transformation

The *BR3* sequence was analyzed as previously described (Jiang et al., 2023). The *BR3* sequence was amplified from ‘Chiifu’ and inserted into the EcoRI and XhoI restriction sites of pBinGlyRed3-35S vector which contains the hygromycin resistance gene. The recombinant vector pBinGlyRed3-35S-BR3 was introduced into *Agrobacterium tumefaciens* GV3101 competent cell using the freeze-thaw method. For the heterologous transformation of Chinese cabbage *BR3* into *Arabidopsis*, the methods were based on those used in previous studies (Jiang et al., 2018; Li et al., 2021; Jiang et al., 2023). The primer pairs used in this study are listed in **Supplementary Table S1**.

### 2.3 Photoperiod, vernalization, and GA_3_ treatments

Plants were cultivated under LD (16-h light/8-h dark photoperiod) or SD (16-h dark/8-h light photoperiod) conditions at approximately 22°C with 65% humidity. For vernalization treatment, germinated WT and *BR3*OE seeds were grown at 4°C for 4 weeks. For GA_3_ treatment, WT and *BR3*OE *Arabidopsis* plants were sprayed with 20 μM GA_3_ solution twice per week until flowering. In control groups of WT and *Arabidopsis BR3*OE plants, an equivalent amount of distilled water was sprayed. Phenotypic investigations were conducted following a previous study (Jiang et al., 2023). At least 15 plants were used for each experiment. After the cotyledons of Chinese cabbage ‘GT-24’ were fully expanded, 500 mg/L GA_3_ was sprayed, and samples were collected 12 h after spraying, with a total of six applications. As a control, ‘GT-24’ was treated with an equal volume of distilled water.

### 2.4 Histochemical GUS assay and subcellular localization analyses

Histochemical GUS staining was performed as previously described (Li et al., 2021). Subcellular localization of the BR3 protein was performed according to the previous method (Jiang et al., 2023). After 24 h of incubation in the dark post-injection, samples were transferred to light conditions for continued incubation. Fluorescence signals were observed 48–72 h post-injection using a laser confocal microscope (Leica SP8, Germany) at excitation wavelengths.

### 2.5 Total RNA isolated, first-strand cDNA synthesis, and qRT-PCR

Total RNA isolated, first-strand cDNA synthesis, and qRT-PCR were conducted according to the methods described in previous studies (Jiang et al., 2018, 2023). The primers used for qRT-PCR analysis are listed in **Supplementary Table S1**.

### 2.6 Statistical analysis

Statistical analysis using Student’s *t*-test or one-way ANOVA was performed using SPSS software (v26). Data are presented as the mean ± standard deviation (SD). Graphs were generated using GraphPad Prism software (v9.2).

## 3 Results

### 3.1 Sequence analysis of *BR3*

The *BR3* (*BraA07003496*) gene sequence was 347 bp and contained two exons and one intron located on the chromosome A07 of Chinese cabbage, encoding 76 amino acids (**Supplementary Figure S1**). A search in the NCBI-CDD conserved domain database showed that BR3 did not have any domains. BR3 was not predicted to function as a transcription factor based on the Plant Transcription Factor Database (TFDB). The Group-based Prediction System (GPS) showed that the BR3 protein lacked kinase activity. No signal peptides, cleavage sites, or transmembrane regions were identified. Structural prediction showed that BR3 consisted of α-helices, extended strands, and random coils, with random coils accounting for 42.11% of the structure. These findings suggest that *BR3* is a novel gene with an unknown function, warranting further investigation to elucidate its role.

### 3.2 *BR3* expression patterns in Chinese cabbage

**Figure 1:**
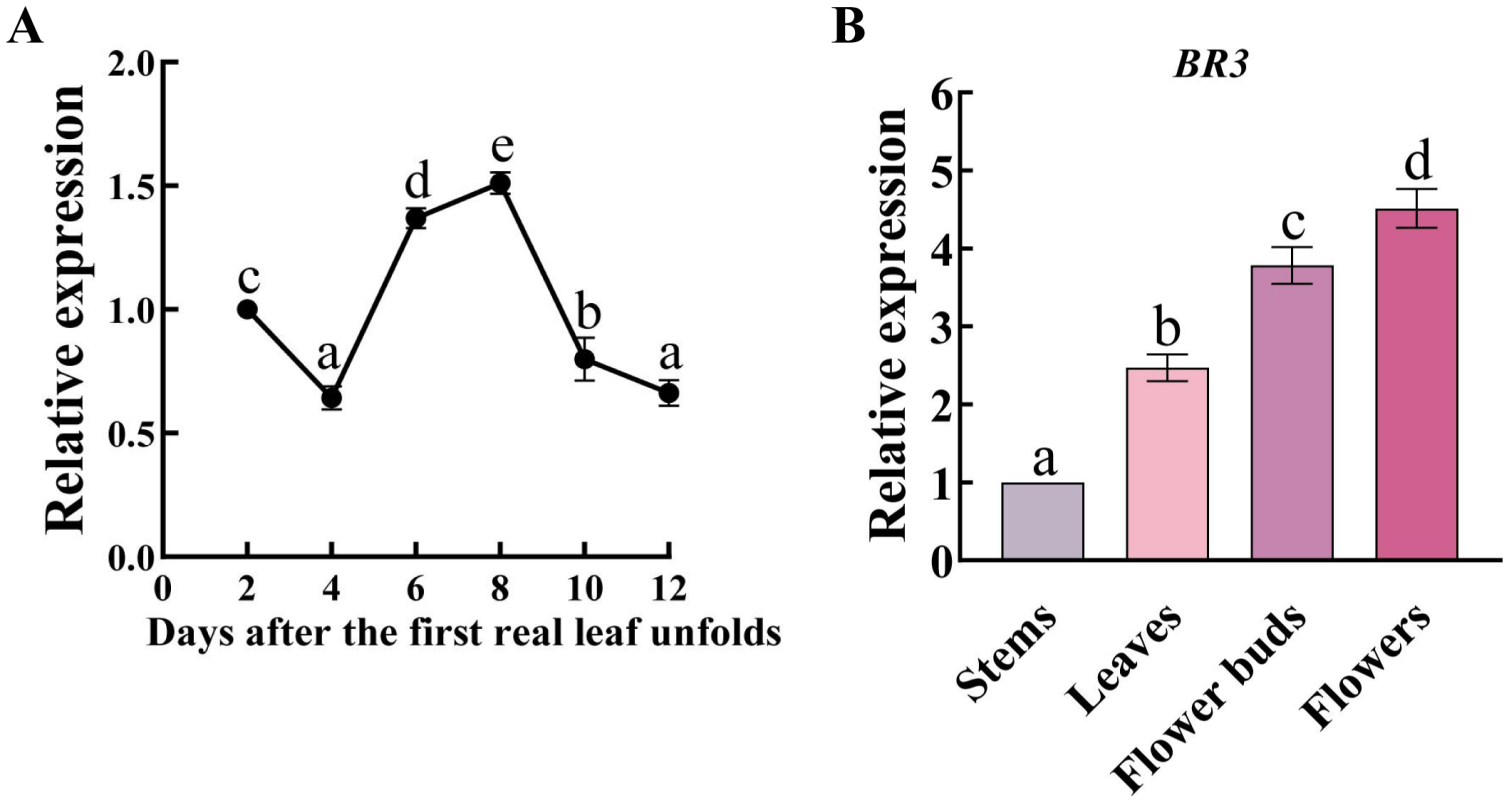
Expression patterns of the *BR3* gene in Chinese cabbage. **(A)** *BR3* gene expression during vegetative stage in Chinese cabbage. The samples of Chinese cabbage ‘GT-24’ cultured under LD conditions were collected from the aboveground parts of the Chinese cabbage at 2, 4, 6, 8, 10, and 12 days after the emergence of the first true leaf. **(B)** *BR3* gene expression during the reproductive stage of Chinese cabbage. The samples were collected from different tissues of the aboveground parts of ‘GT-24’ at 4 days after flowering. There were three biological and three technical replications. Data are presented as the mean ± SD (one-way ANOVA, *p* < 0.05). Different lowercase letters represent significant differences in gene expression between different development stages or tissues.

To investigate the role of *BR3* gene expression during Chinese cabbage development, qRT-PCR analysis was performed on leaves at 2, 4, 6, 8, 10, and 12 days after the emergence of the first true leaf. The gene expression of *BR3* showed notably higher expression levels on days 6 and 8, suggesting that *BR3* gene expression persisted throughout the seedling growth phase (**Figure 1A**). Additionally, at 4 days after flowering, *BR3* expression was detected in the stem, leaf, flower, and flower buds, with the highest expression observed in the flowers (**Figure 1B**). This suggests that *BR3* may directly or indirectly involved in bolting resistance in Chinese cabbage.

### 3.3 *BR3* gene promoter expression analysis and subcellular localization of BR3 protein

**Figure 2:**
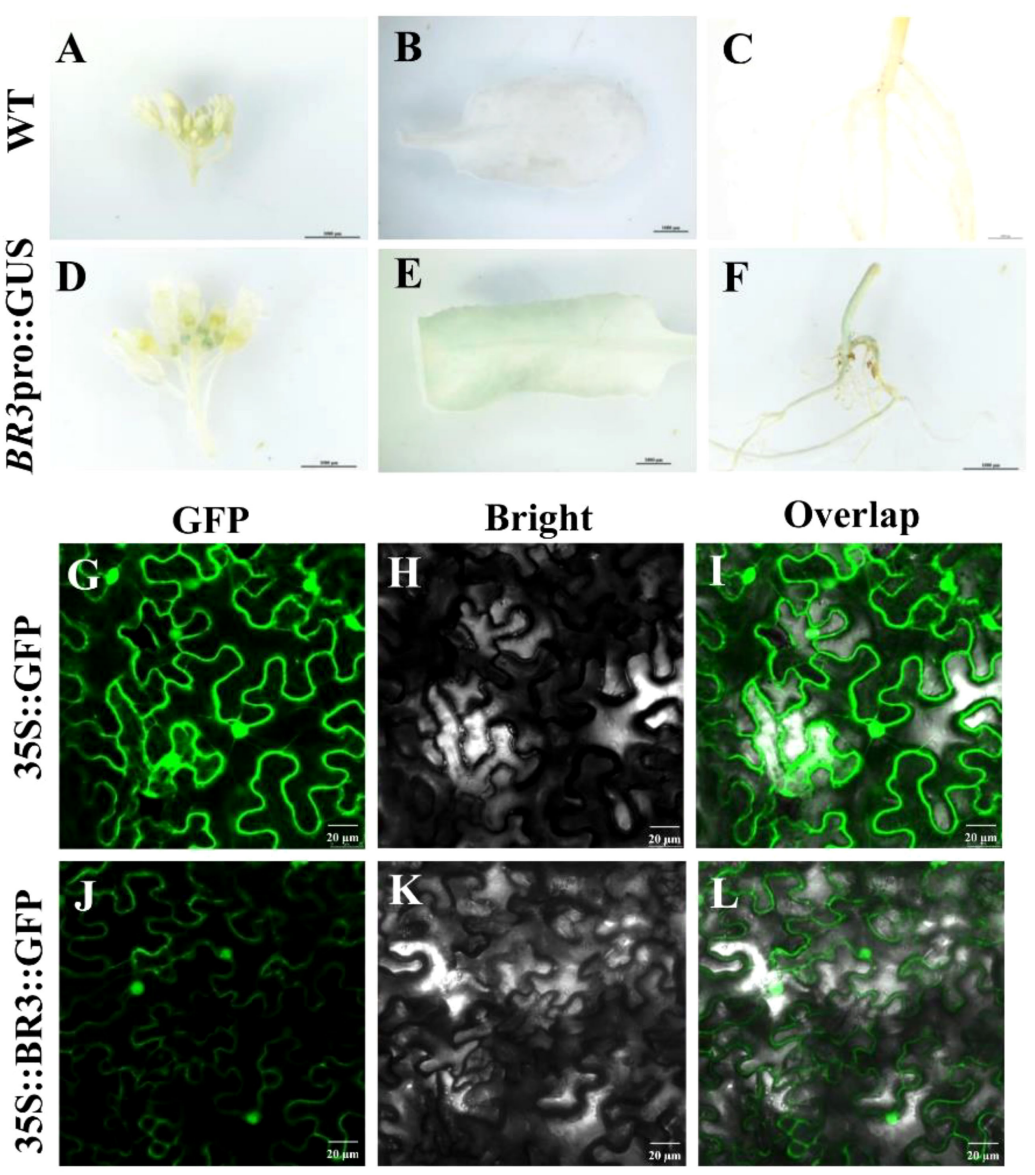
Expression analysis of *BR3* gene promoter and subcellular localization of BR3. **(A-F)** Expression analysis of promoter fusion with GUS. Scale bar: 1000 μm. Subcellular localization of BR3 protein. **(G-I)** 35S::GFP plasmid positive controls and **(J-L)** 35S::BR3::GFP localization in *N. benthamiana*. **(G, J)** GFP fluorescence channels. **(H, K)** Bright field. **(I, L)** Merge field. A Leica confocal microscope was used to collect images at 48 h after agro-infiltration. Control GFP localization was evident throughout these cells. Scale bar: 20 mm.

To determine the spatiotemporal specificity of *BR3* gene expression, GUS staining was performed on the flower buds, leaves, and roots of *BR3* transgenic *Arabidopsis* plants. WT leaves were used as a negative control (**Figures 2A–C**). As shown in **Figures 2D–F**, significant blue staining was observed in the flower buds, leaves, and roots, indicating that the *BR3* gene in *Arabidopsis* is regulated and expressed in these tissues after flowering. To better understand the mechanisms by which the BR3 protein functions within the cell, subcellular localization analysis was conducted. The 35S::BR3::GFP plasmid was introduced into *N. benthamiana* leaves via *Agrobacterium tumefaciens* injection, and fluorescence was observed under a confocal microscope to determine the localization of the BR3 protein. The distribution of fluorescent signals from the transiently expressed fusion protein reveled that BR3 was localized in both the nucleus and plasma membrane (**Figures 2G–L**). These findings provide a foundation for unraveling the flowering regulatory mechanisms of *BR3*.

### 3.4 Delayed flowering of *BR3*OE is independent of photoperiod

To determine whether the late flowering phenotype of *BR3*OE was related to the photoperiod pathway, the flowering times of WT and *BR3*OE plants were recorded under LD and SD conditions.

**Figure 3:**
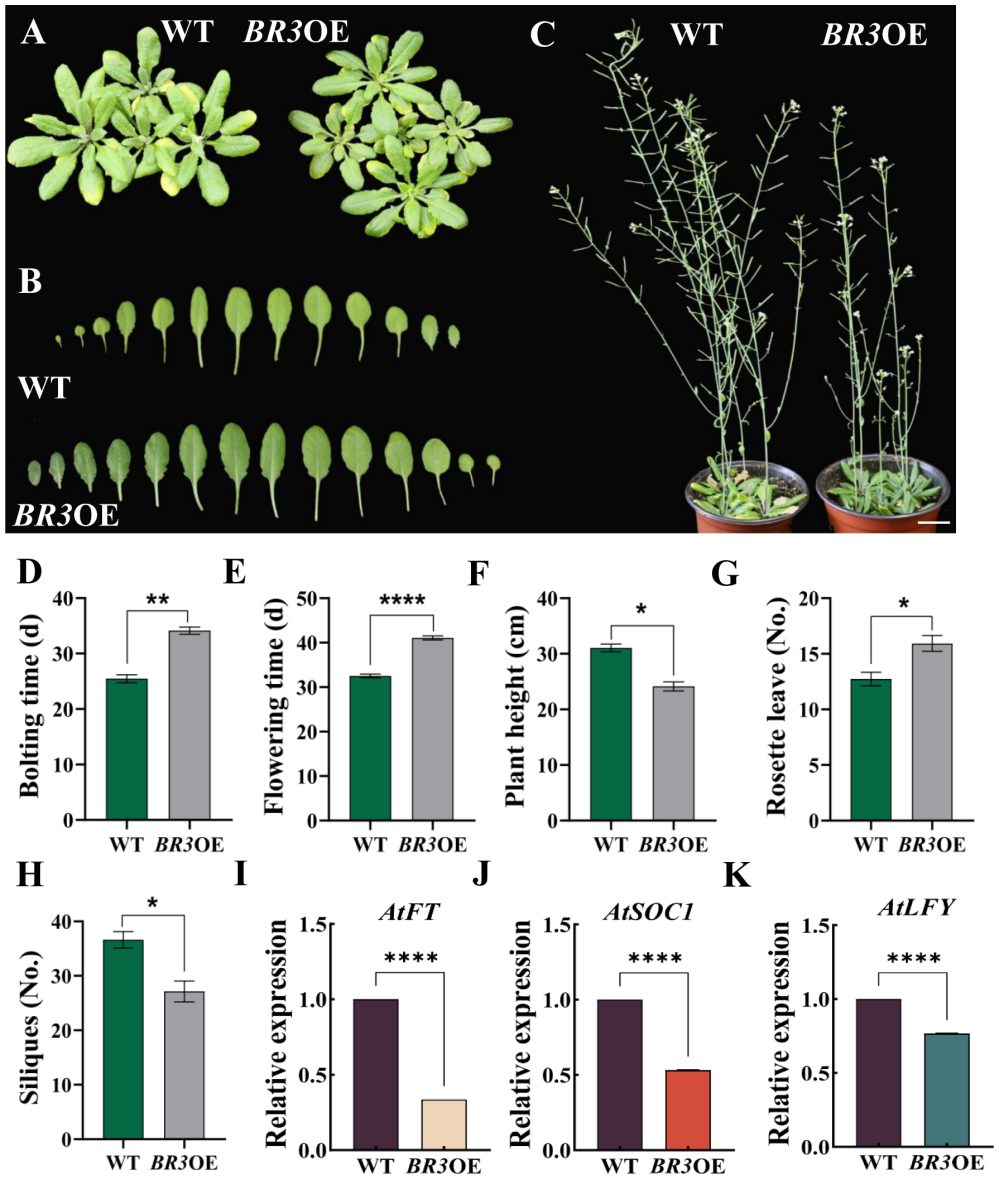
Phenotypes of WT and *BR3*OE under LD conditions and expression of key flowering genes. **(A)** Plant phenotypes of WT and *BR3*OE at 28 days. **(B)** Individual leaves of WT and *BR3*OE at 28 days. **(C)** Plant height of WT and *BR3*OE at 53 days. The scale bars are 2 cm. **(D)** Bolting time, **(E)** flowering time, **(F)** plant height, **(G)** number of rosette leaves, and **(H)** number of siliques of WT and *BR3*OE under LD conditions. **(I-K)** Expression of key flowering genes. Data are presented as the mean ± SD (Student’s *t*-test, ^*^ *p* < 0.05, ^**^ *p* < 0.01, and ^****^ *p* < 0.0001).

*BR3*OE and WT plant phenotypes under LD conditions are shown in **Figures 3A–C**. The bolting time of *BR3*OE plants was 8.66 days later than that of WT (**Figure 3D**). In *BR3*OE plants, flowering time was delayed by 8.53 days (**Figure 3E**), and plant height decreased by 6.92 cm (**Figure 3F**). Concomitantly, the number of rosette leaves increased by 3.2 (**Figure 3G**), and the number of siliques was reduced by 9.47 (**Figure 3H**). Moreover, the phenotype of another *BR3*OE#2 line (**Supplementary Figure S2**) is consistent with that shown in the **Figure 3C**. Then, the expression levels of key flowering genes *AtFT*, *AtSOC1*, and *AtLFY* were measured using qRT-PCR. As shown in **Figures 3I–K**, the expression levels of *AtFT*, *AtSOC1*, and *AtLFY* were significantly downregulated in *BR3*OE plants compared with WT. These results suggest that *BR3* delays flowering in *Arabidopsis* by repressing the expression of *AtFT*, *AtSOC1*, and *AtLFY*.

**Figure 4:**
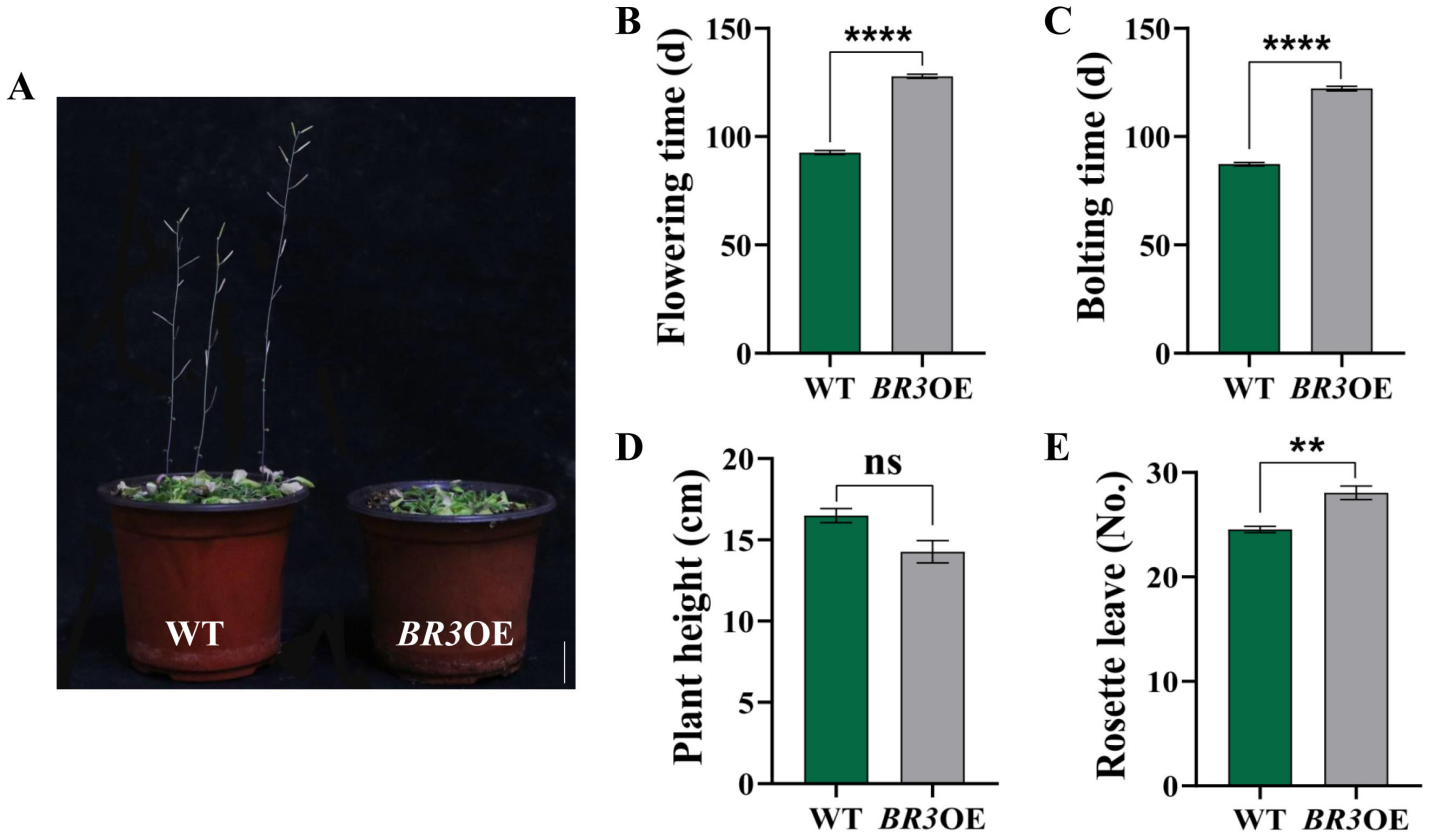
Phenotypes of WT and *BR3*OE plants under SD. **(A)** Phenotypes of WT and *BR3*OE at 120 days. The scale bars are 2 cm. **(B)** Bolting time, **(C)** flowering time, **(D)** plant height, and **(E)** number of rosette leaves of WT and *BR3*OE plants. Data are presented as the mean ± SD (Student’s *t*-test, ^**^ *p* < 0.01, and ^****^ *p* < 0.0001).

The growth phenotypes of *BR3*OE and WT plants under SD conditions are shown in **Figure 4A**. The bolting time was delayed by 34.87 days (**Figure 4B**), and the flowering time of *BR3*OE plants was delayed by 35.27 days compared with that of WT plants (**Figure 4C**). The plant height was reduced by 2.21 cm (**Figure 4D**), and the number of rosette leaves increased by 3.53 (**Figure 4E**). *BR3* gene overexpression led to a late-flowering phenotype under both LD and SD conditions, suggesting that delayed flowering in *BR3*OE is not influenced by the photoperiod. Additionally, the increased number of rosette leaves in *BR3*OE plants at the time of flowering suggests that *BR3* promotes biomass accumulation, enhancing vegetative growth and inhibiting reproductive growth in *Arabidopsis*. These results indicate that *BR3* regulates bolting resistance independent of the photoperiod.

### 3.5 *BR3* delays flowering independent of the vernalization pathway

**Figure 5:**
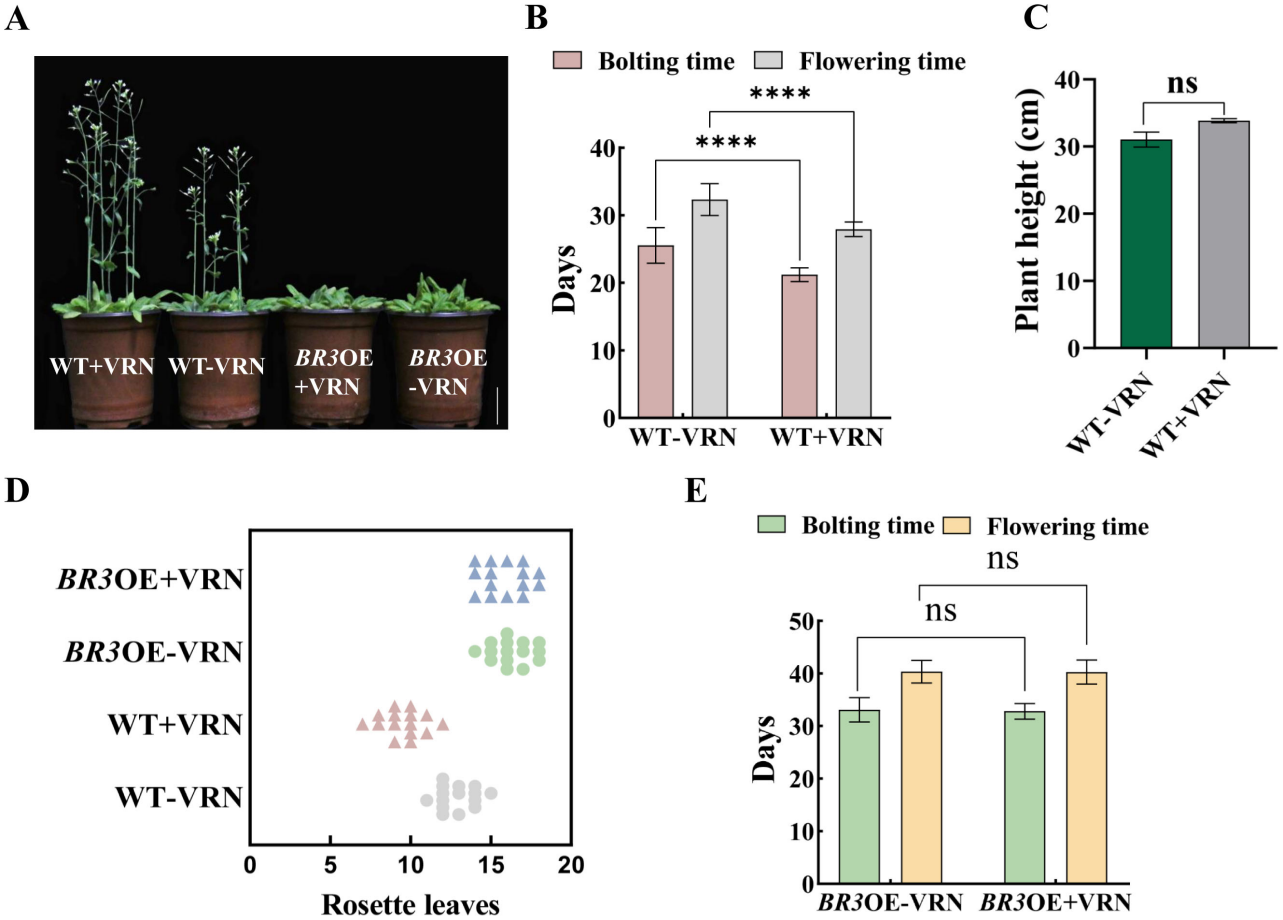
Agronomic traits in WT and *BR3*OE plants after vernalization treatment. **(A)** Phenotypes of WT and *BR3*OE control plants and plants treated with vernalization at 36 days. The scale bars are 2 cm. **(B)** Bolting and flowering time, **(C)** plant height, and **(D)** number of rosette leaves of vernalized and non-vernalized *Arabidopsis* WT plants. **(E)** Bolting and flowering time of vernalized and non-vernalized *Arabidopsis BR3*OE plants. +VRN, With vernalization treatment. -VRN, Without vernalization treatment. Data are presented as the mean ± SD; ns indicates not significant (Student’s *t*-test, ^****^ *p* < 0.0001).

The bolting and flowering times of WT were advanced by 4.33 and 4.4 days, respectively, after vernalization treatment (**Figures 5A, B**). Additionally, compared with the non-treated group, plant height increased by 2.8 cm, and the number of rosette leaves was reduced by 3.2 (**Figures 5C, D**). However, in vernalized *BR3*OE plants, there were no significant differences in bolting time, flowering time, or number of rosette leaves (**Figures 5D, E**). These results indicate that vernalization promotes early flowering in WT but not in *BR3*OE plants, suggesting that the *BR3* gene delays flowering independently of the vernalization pathway and may function through other pathways.

### 3.6 *BR3*OE is responsive to the GA pathway

**Figure 6:**
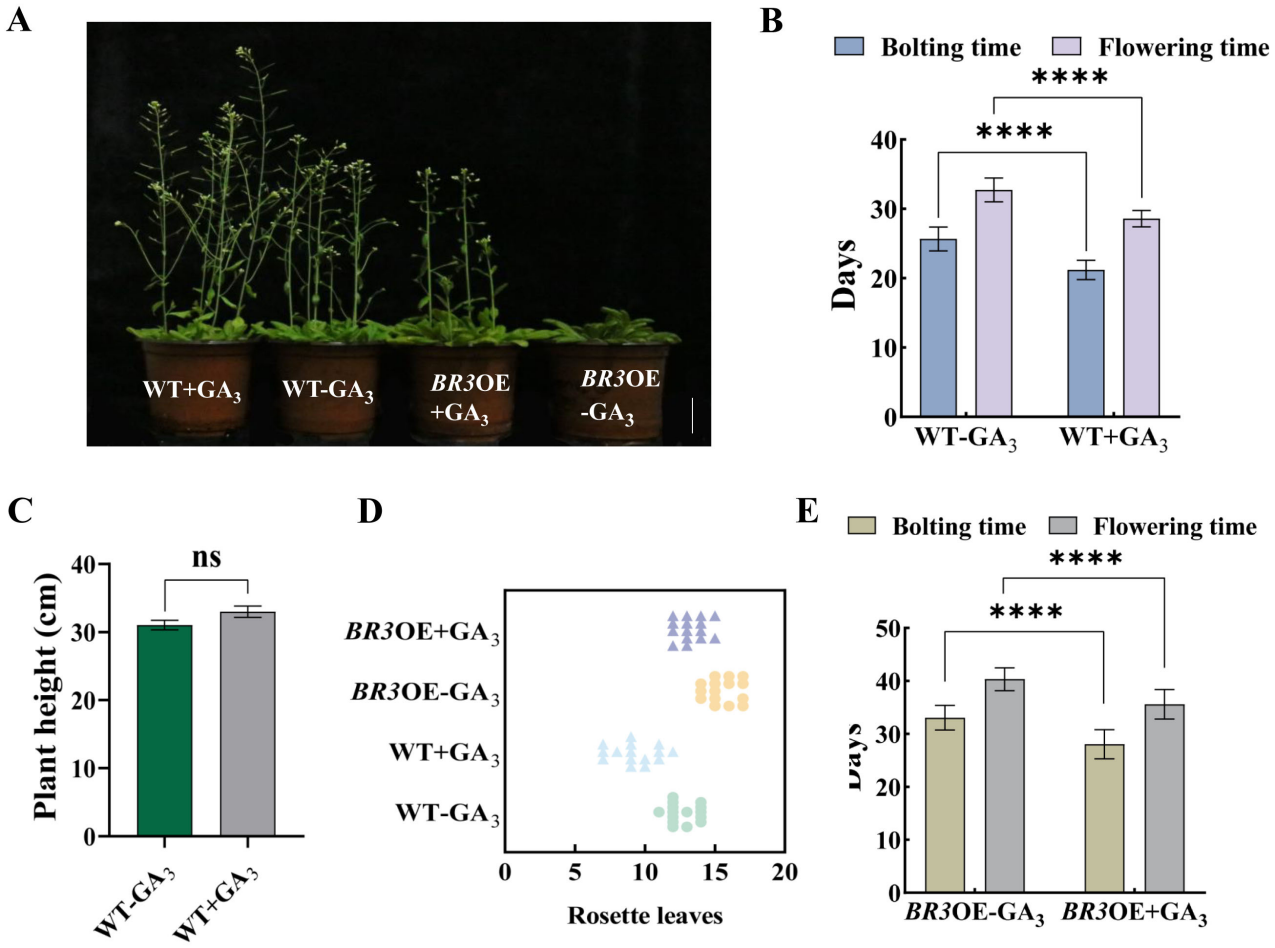
Agronomic traits in WT and *BR3*OE plants treated with GA_3_. **(A)** Phenotypes of WT and *BR3*OE control plants and plants treated with GA_3_ at 36 days. The scale bars are 2 cm. **(B)** Bolting and flowering time and **(C)** plant height of WT plants after GA_3_ treatment. **(D)** Number of rosette leaves in WT and *BR3*OE plants treated with GA_3_. **(E)** Bolting and flowering times of *BR3*OE plants treated with GA_3_. +GA_3_, With vernalization treatment. -GA_3_, Without vernalization treatment. Data are presented as the mean ± SD (Student’s *t*-test, ^****^ *p* < 0.0001).

**Figure 7:**
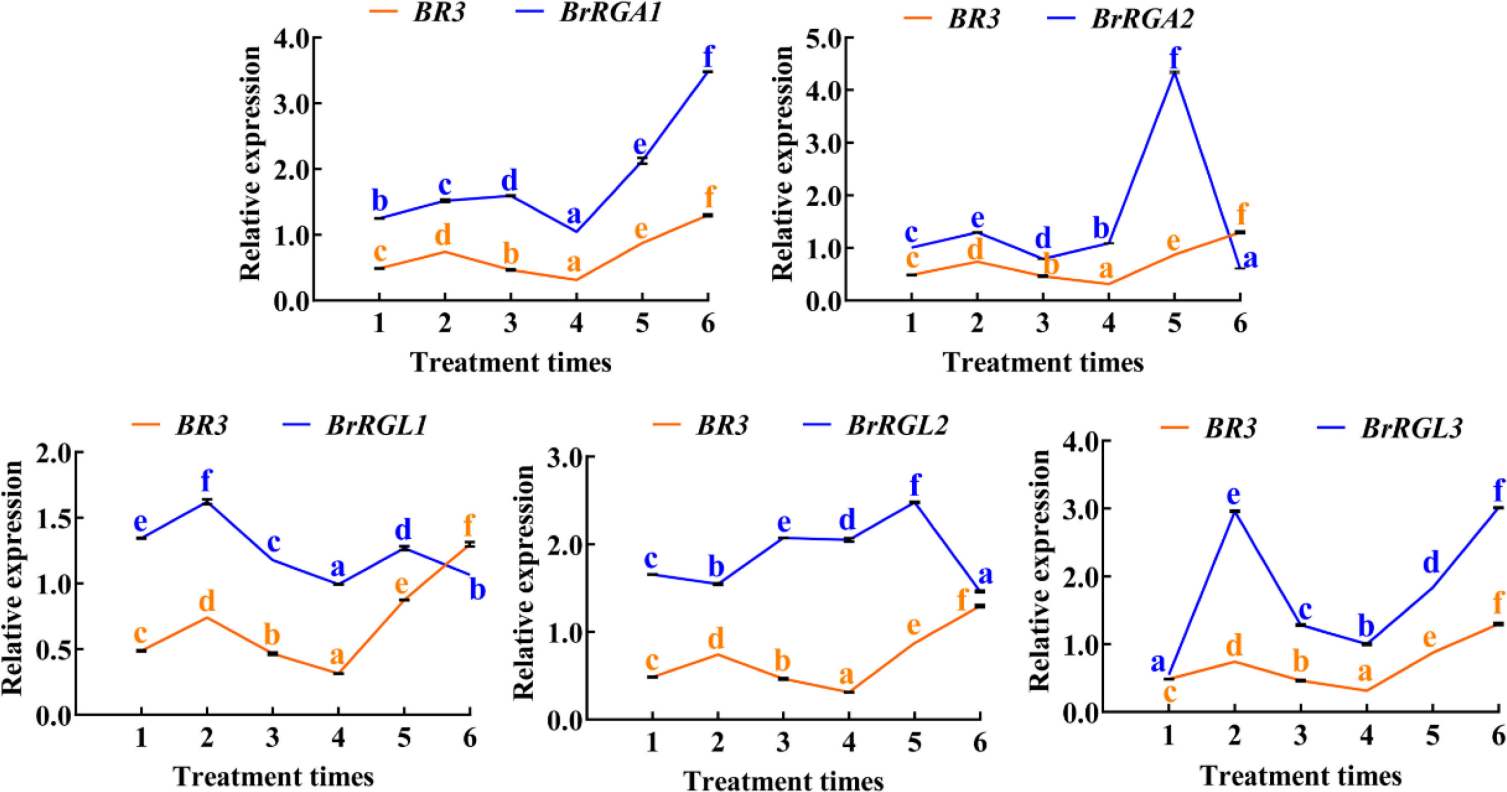
Expression analysis of *BR3* and *DELLA* genes in Chinese cabbage after GA_3_ treatment*. RGA1*, *REPRESSOR of GA1*; *RGA2*, *REPRESSOR of GA2*; *RGL1*, *RGA-LIKE PROTEIN 3*; *RGL2*, *RGA-LIKE PROTEIN 2*; and *RGL3*, *RGA-LIKE PROTEIN 3*. The blue and yellow solid lines represent qRT-PCR results of the *DELLA* and *BR3* genes of Chinese cabbage ‘GT-24’, respectively. Data are presented as the mean ± SD (one-way ANOVA, *p* < 0.05). Different lowercase letters represent significant differences in gene expression between different treatment times.

After treatment with GA_3_, the bolting and flowering times of WT were advanced by 4.47 and 4.13 days, respectively, compared with the non-treated control group (**Figures 6A, B**). Plant height increased by 1.98 cm, and the number of rosette leaves decreased by 3.47 (**Figures 6C, D**). After GA_3_ treatment, the bolting and flowering times of *BR3*OE plants were advanced by 5 and 4.73 days, respectively (**Figure 6E**), compared with non-treated plants, and the number of rosette leaves was reduced by 2.67 (**Figure 6D**). Exogenous GA_3_ application promoted flowering in *BR3*OE plants, which displayed a phenotype similar to WT (**Figure 6A**), suggesting that the *BR3* gene influences flowering gene pathways in response to GA, leading to delayed flowering. DELLA proteins are key transcription factors in the GA signaling pathway. The *B. rapa* genome contains five DELLA subfamily members: *BrRGL1*, *BrRGL2*, *BrRGL3*, *BrRGA1*, and *BrRGA2*. The expression patterns of the five *DELLA* genes and the *BR3* gene in Chinese cabbage were analyzed using qRT-PCR. The expression of the *BrRGA2*, *BrRGL1*, and *BrRGL2* genes significantly decreased after the fifth sampling point. With the increase in the time and frequency of GA_3_ treatments, the expression of the *BrRGA1* and *BrRGL3* genes significantly increased after the fifth sampling, with a consistent increase in *BR3* expression (**Figure 7**).

## 4 Discussion

*OGs* are unique genes in plant genomes that regulate species-specific development, metabolism, and stress responses, enabling plants to adapt to specific environments, optimize metabolic pathways, and enhance stress resistance. Although the function of most *OGs* remains unknown, these genes are ubiquitously present in all species (Jiang et al., 2020b), highlighting the biological significance of the function and mechanisms of *BrOGs*. Previous studies have screened and identified *OGs* in *B. rapa* and thoroughly analyzed *BrOGs* sequence characteristics and expression patterns (Jiang et al., 2018). This study identified a novel *OG*, *BR3*, which positively regulated bolting tolerance in *Arabidopsis*, which further confirming the relationship between *OGs* and species-specific trait formation. Sequence analysis showed that *BR3* with an unknown function that localized to both the cell membrane and nucleus, and key flowering genes were downregulated in *BR3*OE plants. Similarly, *BR1* overexpression downregulates key flowering integrators, such as *AtSOC1*, *AtLFY*, and *AtFUL* (Jiang et al., 2023). Additionally, *BR2* was found to be a positive regulator of bolting resistance through the vernalization pathway that localizes in the cell membrane, and in vernalized Chinese cabbage *BR2*OE, *BrVIN3.b* and *BrFRI* are downregulated, while *BrFLC5* is upregulated, with key flowering factors, such as *BrSOC1s*, *BrLFYs*, and *BrFTs*, downregulated (Zu et al., 2024). These studies strongly support the findings of this study. *BR3*OE exhibited a bolting-resistant phenotype, and exogenous application of GA_3_ promoted flowering. Therefore, *BR3* might delay flowering by acting on key genes in the GA pathway. The differences in subcellular localization and promoter-induced expression indicate distinct *OGs* that regulate bolting resistance through different pathways.

In this study, *BR3* overexpression resulted in a delayed flowering phenotype under LD conditions (**Figures 3C, E**). Moreover, *BR3*OE plants showed significantly reduced expression of the *AtFT*, *AtSOC1*, and *AtLFY* genes compared with WT plants (**Figures 3I–K**). *AtFT* acts as a central integrator of environmental and endogenous signals that is translated into protein in the leaves and transported to the shoot apical meristem, where it upregulates *AtSOC1* expression (Corbesier et al., 2007). *SOC1* acts as a flowering integrator, coordinating other signaling pathways, such as photoperiod and temperature pathways, to regulate flowering time (Blazquez et al., 1998; Moon et al., 2003; Gregis et al., 2009; Liu et al., 2009; Fornara et al., 2010). *LFY* is a key flowering activator whose high expression promotes floral organ formation. Additionally, *SOC1* enhances *LFY* expression by binding to its promoter region (Weigel et al., 1992). Exogenous GA_3_ application significantly enhances *SOC1* expression in *Arabidopsis*, thereby shortening flowering time (Wang et al., 2022). Furthermore, GA influences flowering timing by directly affecting the expression of flowering regulatory genes such as *LFY* and *SOC1* (Mutasa-Göttgens and Hedden, 2009). Simultaneously, *FT* may also influence GA metabolism by regulating key enzymes, such as GA2 oxidase 8-3 (GA2ox8-3). *FT* overexpression under LD conditions reduces *GA2ox8-3*expression (Miskolczi et al., 2019). Under SD conditions, endogenous GA levels increase significantly before flowering, promoting flowering by inducing FT in the leaves and *SOC1* in the shoot apex (Fukazawa et al., 2021). GA promotes flowering by upregulating *FT* transcription under LD conditions (Hisamatsu and King, 2008; Porri et al., 2012). This suggests that *BR3* may inhibit bolting and flowering in *Arabidopsis* through the GA pathway.

In this study, among *Arabidopsis BR3*OE plants subjected to photoperiod, vernalization, and GA_3_ treatments, only GA_3_-treated plants exhibited early flowering (**Figure 6**). *BR3*OE exhibited a late bolting phenotypes, and GA_3_ treatment promoted bolting and flowering. However, despite GA_3_ treatment, bolting and flowering occurred later in *BR3*OE than in WT plants (**Figures 6A, B, E**). DELLA proteins are negative regulators of the GA signaling pathway and inhibit the expression of flowering-related genes by interfering with transcription factor activities. When GA levels increase, DELLA protein degradation alleviates this repression, promoting flowering gene expression (Achard et al., 2003; Sun and Gubler, 2004). Studies have shown that BrARGL1, a key DELLA protein in Chinese cabbage, suppresses bolting when overexpressed, resulting in significantly reduced expression of GA-regulated proteins (BraGASA6), flowering-related genes (*BraSOC1*, *BraLFY*), expansin proteins (BraEXPA11), and xyloglucan endotransglucosylases (BraXTH3). Conversely, *rgl1* mutants show the opposite phenotype. *BRARGL1* inhibits transcriptional activation of *BRASOC1* on *BRAXTH3* and *BRALFY* genes; however, GA_3_ enhances transcriptional activation of *BraSOC1*, indicating that the *BraRGL1*-*BraSOC1* module regulates bolting and flowering in Chinese cabbage through the GA signaling pathway (Wang et al., 2023). The expression of *BrRGA2*, *BrRGL1*, and *BrRGL2* decreased with increased GA_3_ application, potentially due to their degradation. The expression levels of *BrRGA1* and *BrRGL3* were consistent with *BR3*, suggesting that increased *BR3* expression promotes the of *BrRGA1* and *BrRGL3* expression (**Figure 7**). *BR3* increases bolting resistance by increasing the expression of *DELLA* genes in the GA pathway.

Premature bolting is a primary limiting factor for spring-sown Chinese cabbage and cultivation in high-altitude, cold regions, leading to reduced yield and quality and causing significant economic losses. Therefore, identifying bolting resistance genes and developing bolting-resistant varieties are critical for ensuring a year-round balanced supply and stable production. In this study, *Arabidopsis BR3*OE exhibited bolting resistance. After GA_3_ treatment, bolting and flowering were promoted but occurred later than in GA_3_-treated WT, suggesting that *BR3* may regulate bolting through the GA pathway. However, the proteins interacting with BR3 in Chinese cabbage, the transcription factors regulating its expression, and the molecular mechanisms by which the *BR3* gene controls bolting resistance in Chinese cabbage remain unclear. Addressing these topics will provide a theoretical basis for elucidating the molecular mechanism of bolting resistance and offer new insights and gene resources for breeding bolting-resistant Chinese cabbage varieties.

## 5 Conclusions

In this study, a newly identified *OG*, *BR3*, positively regulated bolting resistance, supporting the role of *OGs* in controlling species-specific trait formation. The *BR3* gene was highly expressed in flower buds and flowers, and the BR3 protein was localized in the nucleus and cell membrane. *BR3*OE exhibited a bolting-resistant phenotype and suppressed the expression of key flowering genes. Exogenous GA_3_ treatment and qRT-PCR analysis of the *DELLA* gene suggest that *BR3* functions as a novel flowering time regulator through the gibberellin pathway. This study provides new insights into the breeding of bolting-resistant Chinese cabbage varieties and provides a theoretical foundation for further research on bolting resistance mechanisms in Chinese cabbage.

## Data Availability

The original contributions presented in the study are included in the article/**Supplementary Material**. Further inquiries can be directed to the corresponding authors.

## Supplementary material

Supplementary Figure 1 Gene structure analysis of *BR3* revealed exons and intron.

Supplementary Figure 2 Phenotypes of WT and additional *BR3*OE#2 lines under LD conditions.
