# Supplementary figures and images for: Chinese cabbage orphan gene BR3 confers bolting resistance to Arabidopsis through the gibberellin pathway
Source: Front Plant Sci. 2025 Jan 20;15:1518962. doi: 10.3389/fpls.2024.1518962 (PMC11788340; doi:10.3389/fpls.2024.1518962)

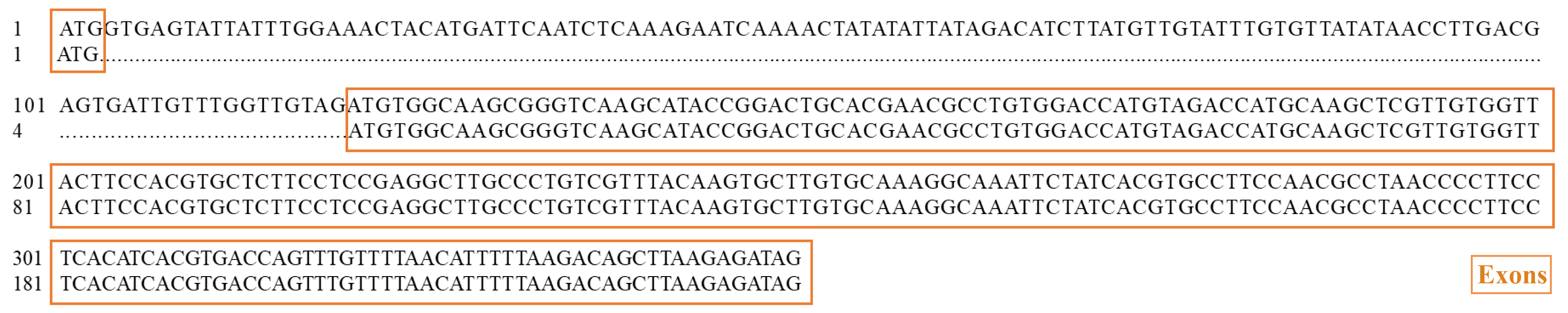

Supplement: Supplementary Figure 1 — Gene structure analysis of BR3 revealed exons and intron. [file Image1.tif]

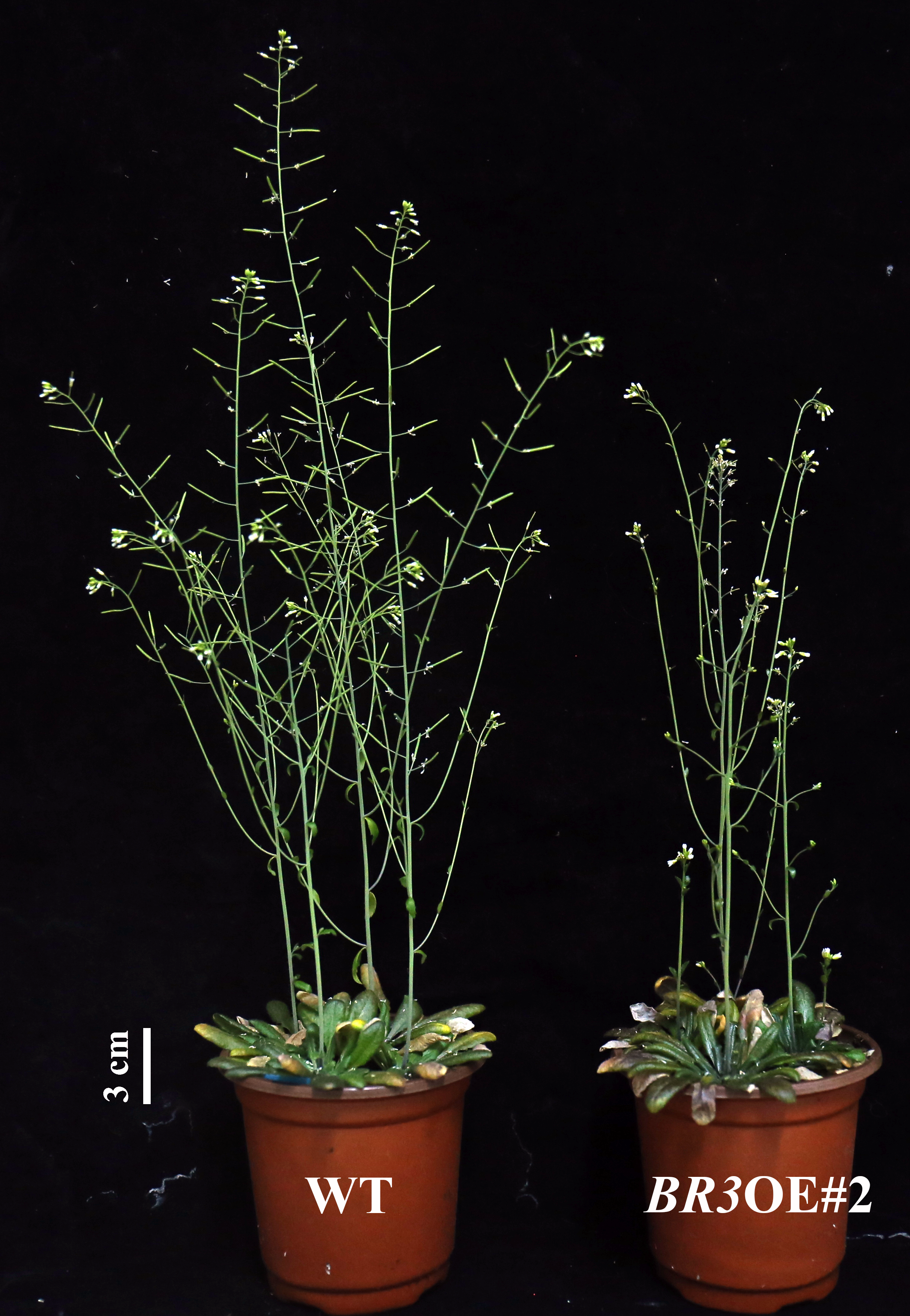

Supplement: Supplementary Figure 2 — Phenotypes of WT and additional BR3OE#2 lines under LD conditions. [file Image2.tif]
